# The Roseibium album (Labrenzia alba) Genome Possesses Multiple Symbiosis Factors Possibly Underpinning Host-Microbe Relationships in the Marine Benthos

**DOI:** 10.1128/MRA.00320-21

**Published:** 2021-08-26

**Authors:** Joana Fernandes Couceiro, Tina Keller-Costa, Matilde Marques, Nikos C. Kyrpides, Tanja Woyke, William B. Whitman, Rodrigo Costa

**Affiliations:** a Institute for Bioengineering and Biosciences, Department of Bioengineering, Instituto Superior Técnico, University of Lisbon, Lisbon, Portugal; b Institute for Health and Bioeconomy Associate Laboratory, Instituto Superior Técnico, University of Lisbon, Lisbon, Portugal; c Department of Energy, Joint Genome Institute, Berkeley, California, USA; d Lawrence Berkeley National Laboratory, Berkeley, California, USA; e Department of Microbiology, University of Georgia, Athens, Georgia, USA; f Centro de Ciências do Mar, University of Algarve, Faro, Portugal; University of Southern California

## Abstract

Here, we announce the genomes of eight Roseibium album (synonym Labrenzia alba) strains that were obtained from the octocoral Eunicella labiata. Genome annotation revealed multiple symbiosis factors common to all genomes, such as eukaryotic-like repeat protein- and multidrug resistance-encoding genes, which likely underpin symbiotic relationships with marine invertebrate hosts.

## ANNOUNCEMENT

**FIG 1:**
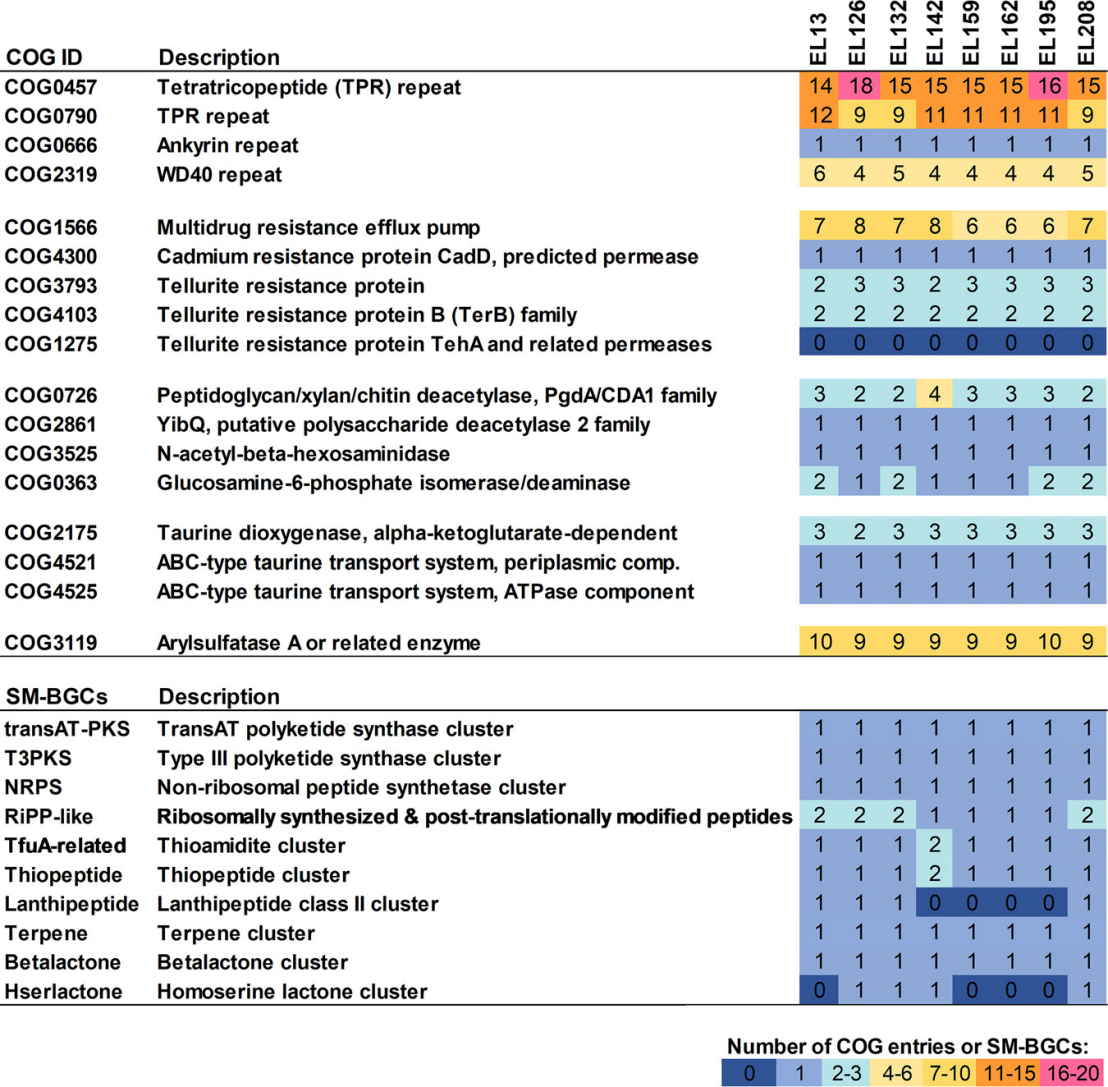
Selected COG functions characteristic of the Roseibium album (Labrenzia alba) genomes described here, as well as SM-BGCs present in all genomes. Values for each entry represent the numbers of coding sequences assigned to COG functions per genome (top) and the numbers of SM-BGCs coding for major compound classes (such as polyketides and terpenes) per genome (bottom).

Roseibium album (1) (homotypic synonyms, Stappia alba [2] and Labrenzia alba [3]) is a Gram-negative, marine alphaproteobacterium (order *Hyphomicrobiales*, family *Stappiaceae*) that has frequently been isolated from sessile, filter-feeding invertebrates such as sponges, corals, and oysters (2, 4–6). To date, however, only a few genomes of this species are available. To illuminate the putative roles of R. album in association with marine animals, here we report the genomes of eight R. album strains that were isolated from the octocoral Eunicella labiata (Table 1), and we present symbiosis factors and environmental resistance traits common to all genomes (Fig. 1).

**TABLE 1:** General features of the Roseibium album (Labrenzia alba) genomes reported in this study

Genome^*a*^	IMG/M identifier^*b*^	Genome size (Mb)	GC content (%)	Genome coverage (×)	No. of contigs	Contig *N*_50_ (bp)	No. of reads	Read length (bp)	Completeness (%)	Contamination (%)	No. of coding sequences	No. of RNAs	No. of genes in COGs	GenBank accession no.	Assembly accession no.	SRA accession no.
Labrenzia alba EL_13	2882936267	7.00	56.44	211.9	19	668,536	19,078,598	151	98.1	2.8	6,475	59	5,303	JADOUQ000000000	GCA_015751945.1	SRR13202144
Labrenzia alba EL_126	2880767578	7.14	56.34	210.4	76	352,437	31,261,630	151	98.1	2.8	6,732	61	5,352	JADOUN000000000	GCA_015752395.1	SRR13202553
Labrenzia alba EL_132	2880560698	7.06	56.44	215.3	51	400,118	33,793,138	151	98.1	2.8	6,629	56	5,334	JADOUI000000000	GCA_015752105.1	SRR13202552
Labrenzia alba EL_142	2880567409	7.29	56.31	207.9	54	363,626	31,961,096	151	98.1	2.8	6,808	66	5,460	JADOUJ000000000	GCA_015752355.1	SRR13202549
Labrenzia alba EL_159	2880774395	6.9	56.44	215.3	18	899,415	32,269,048	151	98.1	2.8	6,384	62	5,230	JADOUO000000000	GCA_015752425.1	SRR13202554
Labrenzia alba EL_162	2880574310	6.9	56.44	214.6	18	899,415	20,727,438	151	98.1	2.8	6,387	62	5,230	JADOUK000000000	GCA_015752365.1	SRR13202550
Labrenzia alba EL_195	2880580784	7.13	56.36	211.6	31	464,795	36,215,300	151	98.1	2.8	6,593	59	5,356	JADOUL000000000	GCA_015752055.1	SRR13202548
Labrenzia alba EL_208	2880780866	7.06	56.44	214.8	49	402,705	22,234,602	151	98.1	2.8	6,638	56	5,334	JADOUP000000000	GCA_015752025.1	SRR13202551

a All strains reported in this study were isolated from the octocoral host *Eunicella labiata*.

b Unique genome identifier at the IMG/M portal.

The eight R. album strains were isolated from E. labiata specimens that had been collected off the coast of Faro, Portugal, after plating of host-derived homogenates on half-strength marine agar medium followed by incubation at 18°C for 4 weeks (4). Genomic DNA was extracted from pure cultures using the Wizard genomic DNA purification kit (Promega, Madison, WI, USA) as described previously (4). The isolates were sequenced at the Joint Genome Institute (JGI) as part of the Genomic Encyclopedia of Type Strains Phase IV (KMG-V) project. Default parameters were used for all software unless otherwise specified. Genome libraries (300 bp) were prepared with the KAPA HyperPrep kit (KAPA Biosystems) and sequenced using the Illumina NovaSeq 6000 platform (S4 flow cell). Raw reads were quality filtered per JGI standard operating practice (SOP) protocol 1061 using BBTools v38.86 (http://bbtools.jgi.doe.gov). Filtered reads were assembled into contigs using SPAdes v3.14.1 (7) with 25, 55, and 95 k-mers, and contigs were annotated using the Integrated Microbial Genomes and Microbiomes (IMG/M) pipeline v5.0.17 (8). We report the Clusters of Orthologous Groups of proteins (COG) profiles obtained for all eight strains using the IMG/M platform (9). Average nucleotide identity (ANI) values obtained on the IMG/M platform (9) for all pairwise combinations among the strains were above 98.3%, supporting the same-species status of the strains. Genome completeness and contamination scores were assessed with the Microbial Genomes Atlas (MiGA) (10) (Table 1). AntiSMASH v6.0 (11) was used to identify secondary metabolite biosynthetic gene clusters (SM-BGCs).

Analysis of the R. album genomes revealed the presence of various COGs important for the establishment of symbiotic relationships, including eukaryotic-like WD40, ankyrin, and tetratricopeptide repeats (Fig. 1). COG functions related to drug (e.g., COG1566) and heavy metal resistance were common to all eight genomes, including traits specific for tellurite resistance that are usually encoded on plasmids (12) (Fig. 1). The eight strains harbor genes for the utilization of chitin, a trait that was previously reported for sponge and coral symbionts (13), as revealed by the presence of polysaccharide deacetylases and exochitinases. Other features included a coding potential for a possible role in sulfur cycling, e.g., through the catabolism of the sulfolipid cerebroside 3-sulfate and of the amino-sulfonic acid taurine, two compounds that are widely synthesized in animal tissue and utilized by bacterial symbionts (14). Moreover, all R. album genomes possess the potential to produce a diverse range of secondary metabolites. Indeed, we found a variety of SM-BGCs encoding terpenes, *trans*-AT-polyketide synthases and type III polyketide synthases, nonribosomal peptide synthetases, and several ribosomally synthesized peptides in this genome pool (Fig. 1).

### Data availability.

The genome sequences of the eight R. album (*L. alba*) strains have been deposited in GenBank by the JGI. Accession numbers are listed in Table 1.
